# Characteristics of the Energetic Igniters Through Integrating Al/NiO Nanolaminates on Cr Film Bridge

**DOI:** 10.1186/s11671-015-1204-9

**Published:** 2015-12-30

**Authors:** YiChao Yan, Wei Shi, HongChuan Jiang, Jie Xiong, WanLi Zhang, Yanrong Li

**Affiliations:** State Key Laboratory of Electronic Thin Films and Integrated Devices, University of Electronic Science and Technology of China, Chengdu, 610054 China

**Keywords:** Al/NiO nanolaminates, (Al/NiO)_n_/Cr igniter, Bilayer thickness, Electrical explosion

## Abstract

The energetic igniters through integrating Al/NiO nanolaminates on Cr film bridges have been investigated in this study. The microstructures demonstrate well-defined geometry and sharp interfaces. The depth profiles of the X-ray photoelectron spectroscopy of Al/NiO nanolaminates annealed at 550 °C with a bilayer thickness of 250 nm show that the interdiffusion between the Al layer and NiO layer has happened and the annealing temperature cannot provide enough energy to make the diffusion process much more complete. The electrical explosion characteristics employing a capacitor discharge firing set at the optimized charging voltage of 40 V show that the flame duration time is about 700 μs, and an excellent explosion performance is obtained for (Al/NiO)_n_/Cr igniters with a bilayer thickness of 1000 nm.

## Background

The energetic igniters have attracted much attention in recent years because of lower ignition energy, faster ignition time, and smaller dimensions in volume compared with conventional hot-wire devices, especially for doped polycrystalline silicon [1–5], platinum [6], titanium [7], chromium [8], and tantalum nitride bridges [9]. Nevertheless, this kind of energetic igniters has some disadvantages such as relatively low transient ignition temperature and output energy. A variety of energetic nanolaminates consist of alternating nanoscale layers of metal or metal oxide such as Al/Ni [10–13], B/Ti [14, 15], Al/CuO [16–20], and Al/MoO_x_ [21], which can provide large negative reaction heats. The reaction heat and burning rate are mainly determined by the ingredient and different bilayer thicknesses with different contact surface areas or volume ratios between alternate layers. Integrating nanolaminates on a film ignition bridge has proved to be an effective way to improve the performance, where a self-propagating exothermic reaction can be initiated in these nanolaminates with generated thermal plasma when the applied current passes through a film bridge. The integrated structure combines the advantages of the film bridge and reactive multilayer films, which can optimize the ignition performance with low electrical energy consumption, fast energy release rate, and a large amount of reaction heat.

Chromium can be a promising energetic material for its good physical properties including high stability, good reliability, and large temperature coefficient of resistance (TCR). Among all of the nanolaminates, it is found in the literature [22] that the released reaction heat in Al/NiO nanolaminates is about 3440 J/g and relatively much higher than that of others. In this study, an energetic igniter by integrating Al/NiO nanolaminates on a Cr film bridge is fabricated and characterized. The elemental diffusion process and solid state reaction mechanism in nanolaminates are systematically investigated, and the electrical explosion properties of (Al/NiO)_n_/Cr igniters are also studied.

## Methods

**Fig. 1:**
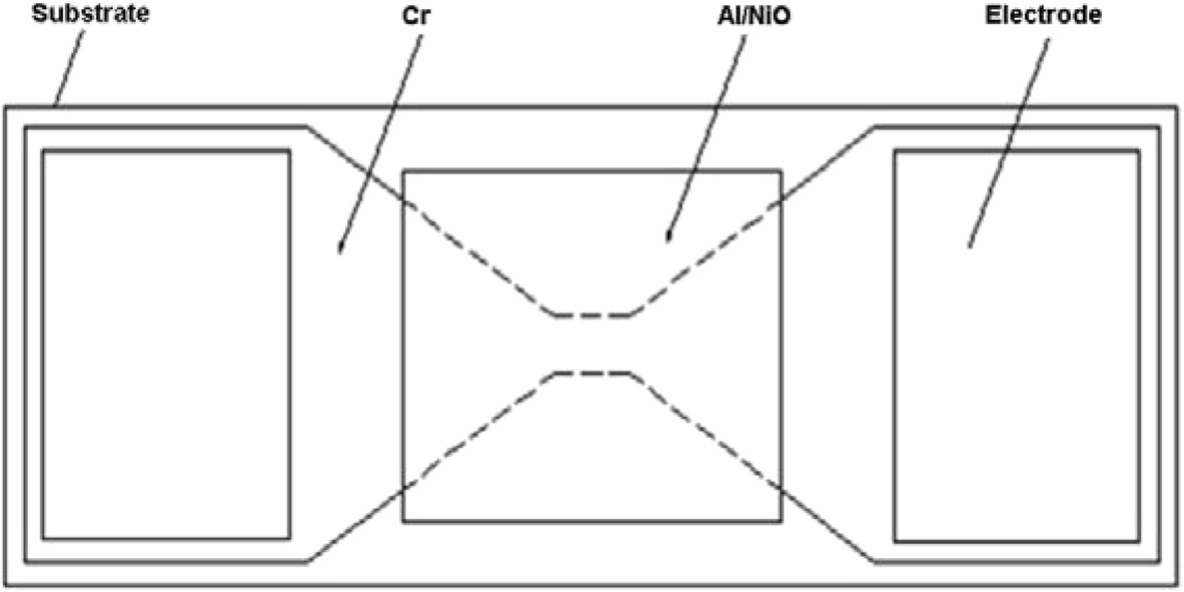
Schematic diagram of an igniter by integrating Al/NiO reactive multilayer films on Cr film bridge

A schematic diagram of (Al/NiO)_n_/Cr igniters is shown in Fig. 1, which consists of an “H”-shaped Cr metal film with wet-etching process, subsequent square-shape (Al/NiO)_n_ nanolaminates with a mask of 4 mm × 4 mm, and two lands of copper electrode. The dimensions of the bridge are 80 μm long (*l*) by 40 μm wide (*w*) by 2 μm thick (*t*).

Cr films are deposited onto alumina substrates (10 mm × 5 mm × 0.5 mm) by direct current (DC) magnetron sputtering. Before deposition, the substrates are cleaned with acetone, alcohol, and de-ionized water in an ultrasonic bath for 10 min, which subsequently are dried by nitrogen gas and placed in the oven for 1.5 h. The distance between the chromium target (99.99 % purity) and alumina substrate is 70 mm to obtain a homogeneous deposited film. When the base pressure is pumped down to 5 × 10^−4^ Pa, argon gas is firstly introduced into the chamber as work gas and then the Cr film is deposited for 1.5 h with a sputtering pressure and power of 0.9 Pa and 128 W, respectively. The spin-coated photoresist (AZ9260) on the Cr film is patterned using photolithography with the designed mask. Subsequently, the exposed Cr film is directly wet-etched in the corrosive liquid and then placed in the oven at 150 °C for 5 min.

Al/NiO nanolaminates are alternately deposited from Al (99.99 % purity) and Ni (99.95 % purity) targets of diameter 50 mm through a shield with an optimized shape by DC magnetron sputtering and DC reactive magnetron sputtering at room temperature, respectively. The distance between the target and substrate is 60 mm with the sputtering power at 100 W for the Al and Ni targets. After each NiO monolayer is obtained with argon partial pressure at 1.5 Pa and oxygen partial pressure at 0.25 Pa, the argon gas and oxygen gas in the chamber are pumped out completely to prevent the oxidation of deposited metal aluminum with argon partial pressure at 1.5 Pa. The deposition rates of Al and NiO monolayers are calculated by dividing the monolayer thickness by the deposition time. The total thickness of Al/NiO nanolaminates is 3 μm, and the thickness ratio of the Al monolayer and NiO monolayer is maintained at 1:1.5 in order to react completely between the Al and NiO films. The resistivity value of the (Al/NiO)_n_/Cr igniter is about 2 Ω. The nanolaminates with bilayer thicknesses of 250 and 1000 nm are fabricated in this paper with the NiO monolayer on the bottom and the Al monolayer on the top.

The cross-sectional and top-view morphologies are determined using a scanning electronic microscopy (SEM, JEOL-7500F). The elemental distribution through the thickness of several layers during the diffusion process in the nanolaminates after post-deposition annealed at 550 °C in flow argon gas is confirmed using X-ray photoelectron spectroscopy (XPS) depth profiling with the interval time of 5 min (Axis Ultra DLD, Kratos Analytical Ltd). The electrical explosion parameters including ignition voltage, ignition current, and ignition delay time are achieved using a capacitor voltage discharging firing set, and the literatures [23–25] describe the principle of open-air electrical explosion testing of the igniter; the explosion process is observed using a high-speed camera (HS4540MX12) with 20,000 frames per second. Component analysis of the corresponding products after electrical explosion experiment is identified using XPS.

## Results and Discussion

**Fig. 2:**
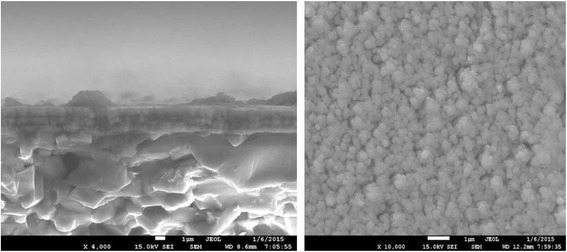
The cross-sectional (*left*) and top-view (*right*) SEM morphologies of Al/NiO nanolaminates with bilayer thickness of 1000 nm

The cross-sectional and top-view SEM morphologies of the as-deposited Al/NiO nanolaminates with a bilayer thickness of 1000 nm are shown in Fig. 2. It is clearly visible that there are well-defined interfaces for the layered structure in Fig. 2. The left image in Fig. 2 provides an obvious thin Al_2_O_3_ oxide layer on the top of the Al monolayer, which indicates that the redox reaction between Al and NiO films has not happened because of a much lower deposition temperature. Although the grains are smaller and equiaxed in the right image, many holes exist on the top of the nanolaminates and the diameters of grains are not uniform due to the oxidized metal Al surface.

**Fig. 3:**
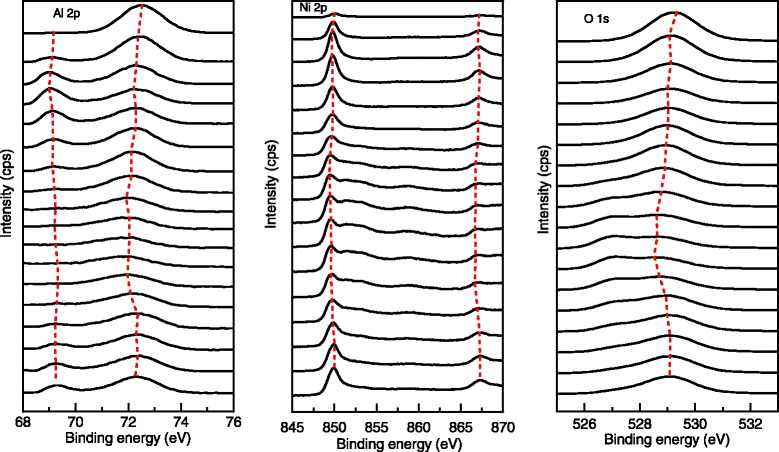
The XPS depth profiles of Al/NiO nanolaminates annealed at 550 °C with bilayer thickness of 250 nm. The *curve on the top* of each image is the first deposited material, and the *bottom curve* is the surface-oxidized metal Al in Al/NiO nanolaminates

In order to confirm the composition and chemical bonds after annealing at 550 °C, XPS depth profiles are obtained by scanning the surface after every 5-min etching. The XPS depth profiles in Fig. 3 provide convincing evidence that oxygen moves out of the NiO and into the Al layer after Al/NiO nanolaminates are annealed at 550 °C. At the beginning of etching, the photoelectron spectra of Al 2p and O 1s have strong intensities, verifying the existence of the Al_2_O_3_ thin layer because of the oxidized metal Al for as-deposited Al/NiO nanolaminates. At the same time, it is also observed that there is an obvious signal of the Ni element on the surface in Fig. 3 due to the strong diffusion ability of the element nickel with the enhanced annealing temperature. As the etching time increases, the intensities of the Al 2p and O 1s photoelectron spectra gradually reduce and a weak peak of metal Al appears, which indicates that the redox reaction between Al and NiO films has not reacted completely because of low input energy annealed at 550 °C. There exists the interfacial transition zone which contains Al, Ni, and O elements within the windows between 70- and 85-min etching. As the etching time increases, the peak positions of Al 2p, Ni 2p, and O 1s move toward high binding energy and the trend is specially obvious when approaching to the interface between the Al layer and Ni layer.

**Fig. 4:**
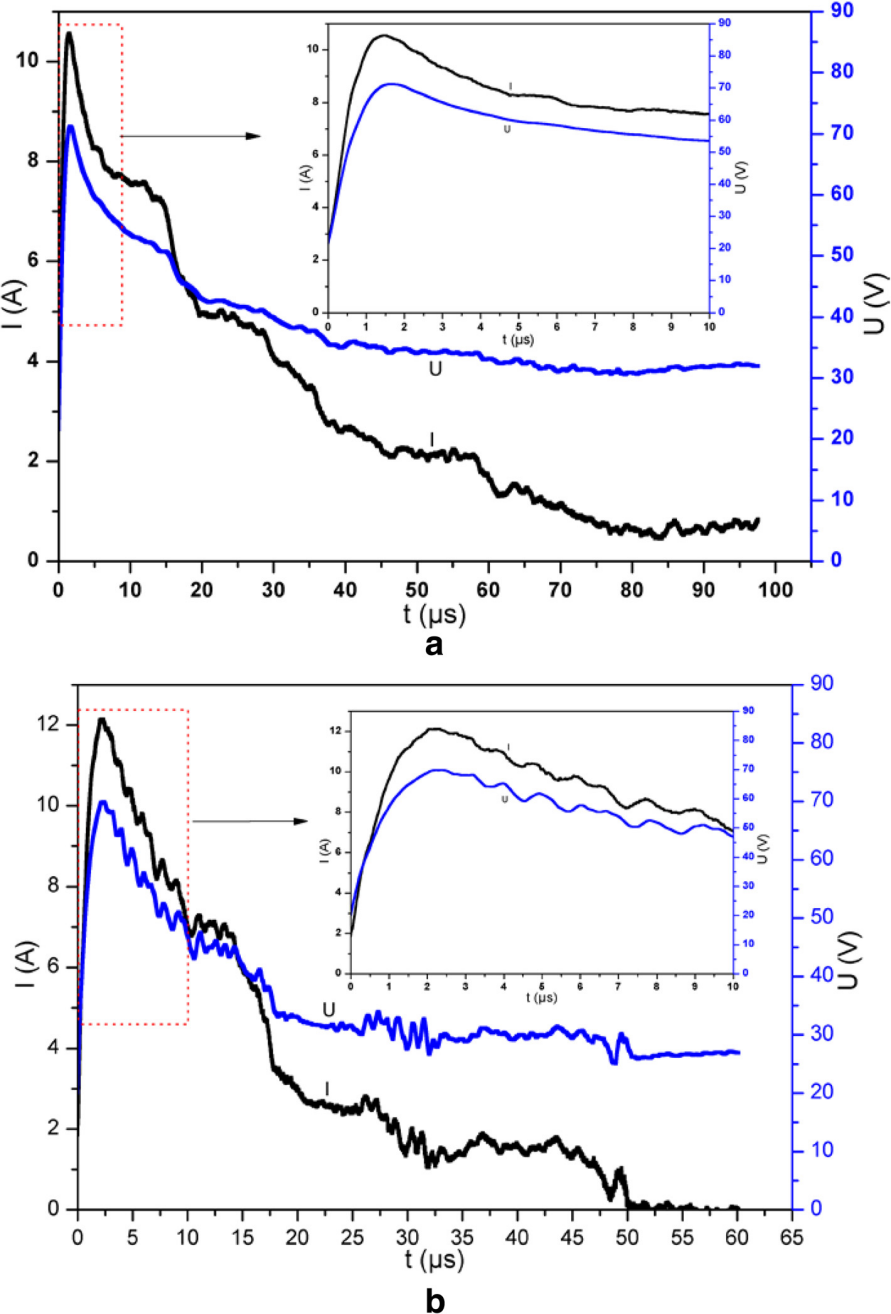
Voltage-current histories for (Al/NiO)_n_/Cr igniters. **a** Bilayer thickness of 1000 nm. **b** Bilayer thickness of 250 nm. The *insets* show partial enlarged details of the selected areas

**Fig. 5:**
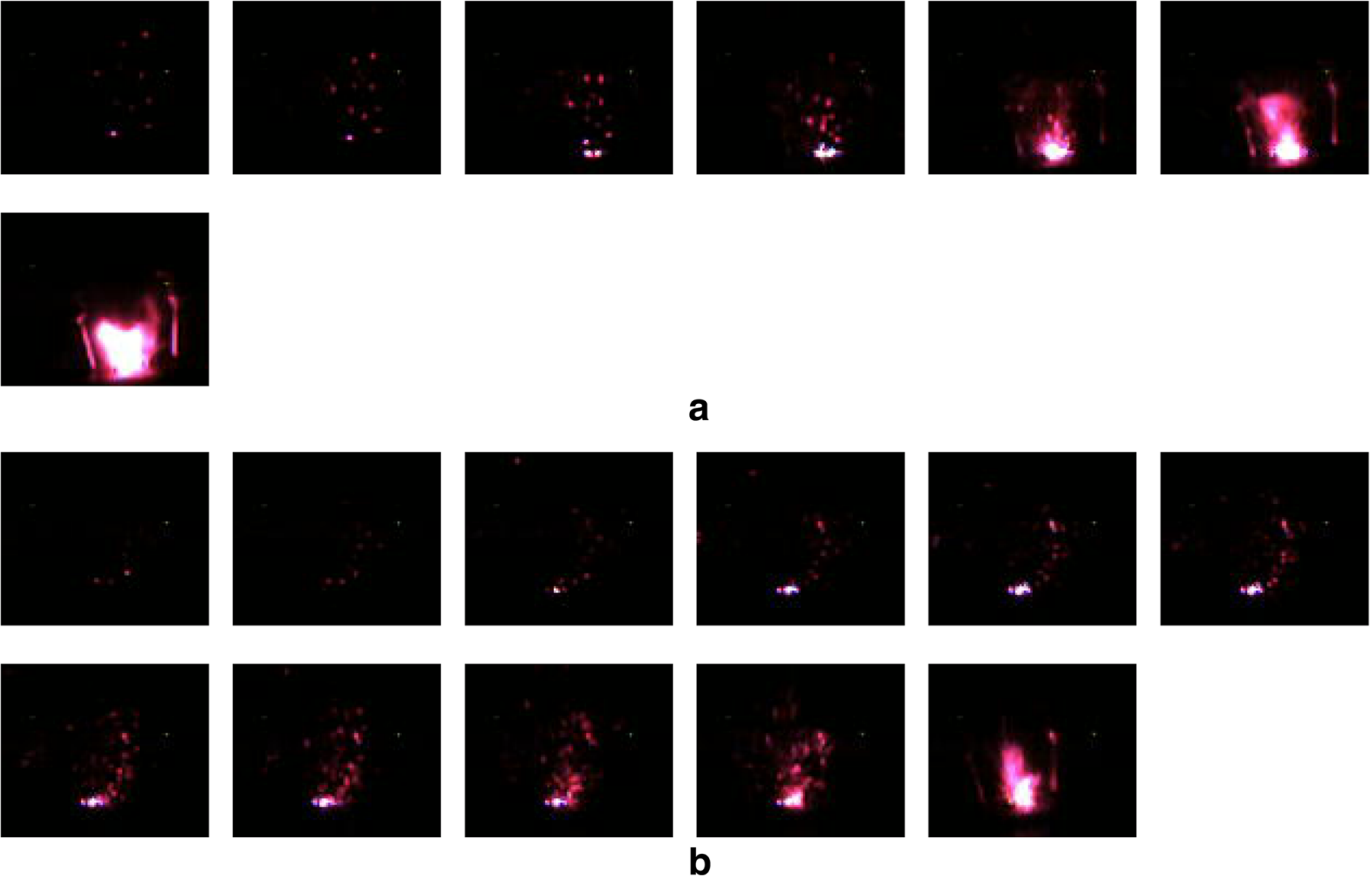
High-speed camera observation of the electrical explosion process for (Al/NiO)_n_/Cr igniters with an interval time of 100 μs between adjoining images. **a** Bilayer thickness of 1000 nm. **b** Bilayer thickness of 250 nm

For investigating the electrical explosion characteristics of (Al/NiO)_n_/Cr igniters, a capacitor discharge firing circuit test system (47 μF, 40 V) is adopted to apply the currents across the energetic igniters. The variations of the voltage and current as a function of time for (Al/NiO)_n_/Cr igniters with the capacitor voltage charged to 40 V are showed in Fig. 4, which is the corresponding optimized discharging voltage and at which the voltage and current curves almost reach the peak simultaneously. Due to the existence of the NiO layer as an electrical insulation layer between the Al layer and Cr layer, the applied current should pass through the Cr film bridge completely. By the comparison of voltage-current curves of (Al/NiO)_n_/Cr with bilayer thicknesses of 1000 and 250 nm, the time of reaching the peak value of voltage for (Al/NiO)_n_/Cr with a bilayer thickness of 1000 nm precedes over that of (Al/NiO)_n_/Cr with a bilayer thickness of 250 nm, which indicates that the amount of thermal boundary conductance across the interfaces increases as the bilayer thickness decreases and the thermal diffusion time becomes greater significantly. The voltage-current histories for (Al/NiO)_n_/Cr igniters are consistent with high-speed camera observations of the electrical explosion process as shown in Fig. 5. The flame duration time of (Al/NiO)_n_/Cr with bilayer thickness of 1000 nm is about 700 μs and nearly half as that of (Al/NiO)_n_/Cr with bilayer thickness of 250 nm, which will release more energy in a short time. It is also observed in Fig. 5 that a more fierce explosion for (Al/NiO)_n_/Cr with bilayer thickness of 1000 nm is accompanied with a much brighter flash of light, much larger quantities, and a longer distance for the ejected product particles. Although a smaller bilayer thickness means a much shorter diffusion distance and quicker burning rate, there are more interfaces and it is more likely that the NiO layer and Al layer partially interdiffuse for a much smaller bilayer thickness before the electrical explosion test, which results in a lower energy output and reduction of the burning rate.

**Fig. 6:**
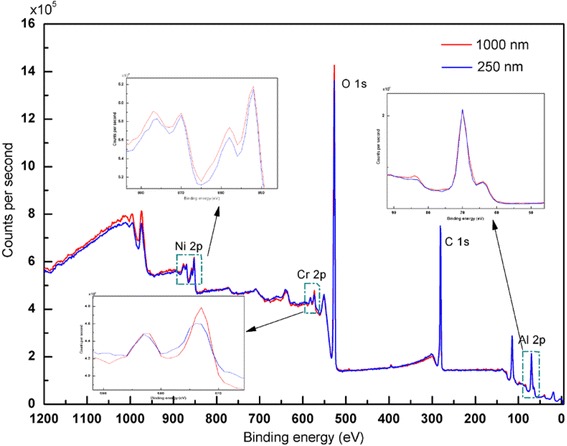
XPS spectra of (Al/NiO)_n_/Cr igniters with bilayer thicknesses of 1000 and 250 nm after the electrical explosion experiment. The *insets* show partial enlarged details of the selected areas

The XPS spectra for the surface region of (Al/NiO)_n_/Cr igniters with bilayer thicknesses of 1000 and 250 nm after the electrical explosion experiment in Fig. 6 strongly reveal the presence of Ni 2p, Cr 2p, O 1s, and Al 2p. There is no remarkable shift for the bilayer thickness from 250 to 1000 nm, while the peak intensity increases obviously correspondingly. The result indicates that the electrical explosion test makes the redox reaction much more complete with a bilayer thickness of 1000 nm, agreeing well with high-speed camera observations of the electrical explosion process in Fig. 5.

## Conclusions

The (Al/NiO)_n_/Cr igniters are fabricated by integrating Al/NiO nanolaminates on the Cr film bridge. The microstructure of Al/NiO nanolaminates with the bilayer thickness of 1000 nm is densely packed and with well-defined interfaces. The XPS depth profiles of Al/NiO nanolaminates annealed at 550 °C with bilayer thickness of 250 nm show that the diffusion between the Al layer and NiO layer has happened and the annealing temperature cannot provide enough energy to make the diffusion process much more complete. The electrical explosion test and XPS spectra exhibit that the (Al/NiO)_n_/Cr igniter with bilayer thickness of 1000 nm has more advantages over that of 250 nm. The (Al/NiO)_n_/Cr igniter is supposed to have a variety of potential applications in both military and civilian areas, and it should be noted that it allows batch fabrication and a high level of integration by standard microfabrication techniques.
